# Supplementary figures and images for: Client processing is altered by novel myopathy-causing mutations in the HSP40 J domain
Source: PLoS One. 2020 Jun 4;15(6):e0234207. doi: 10.1371/journal.pone.0234207 (PMC7272046; doi:10.1371/journal.pone.0234207)

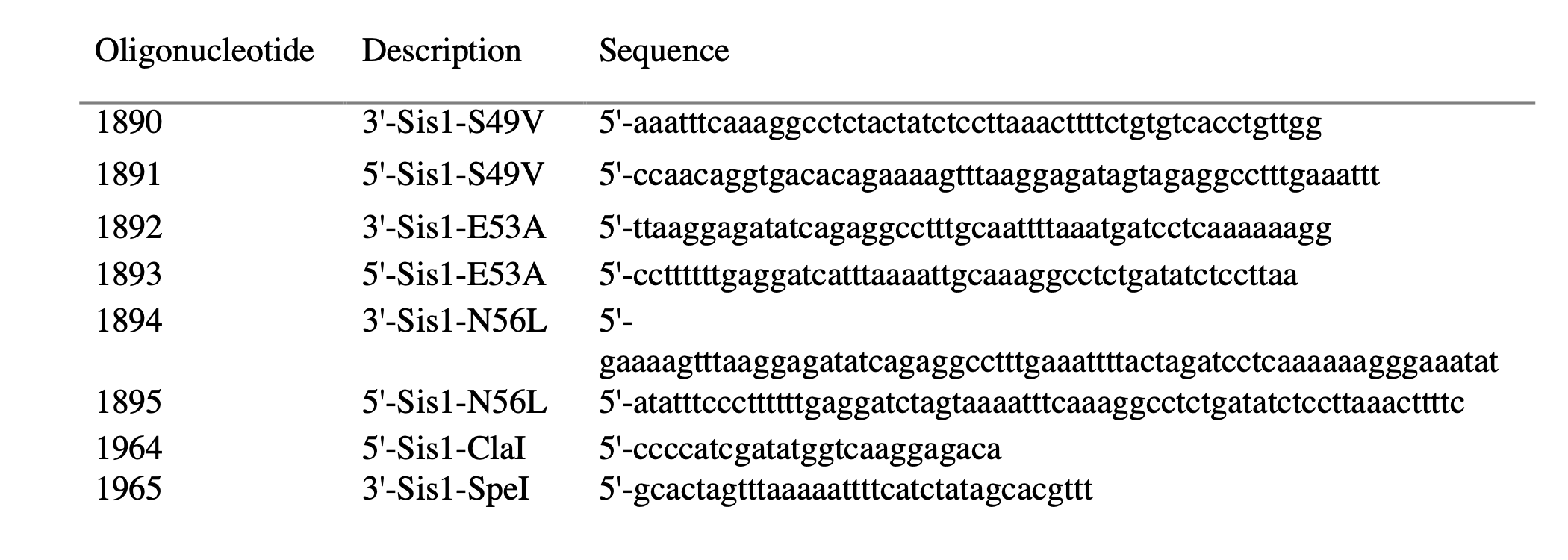

Supplement: S1 Table — (TIF) [file pone.0234207.s001.tif]

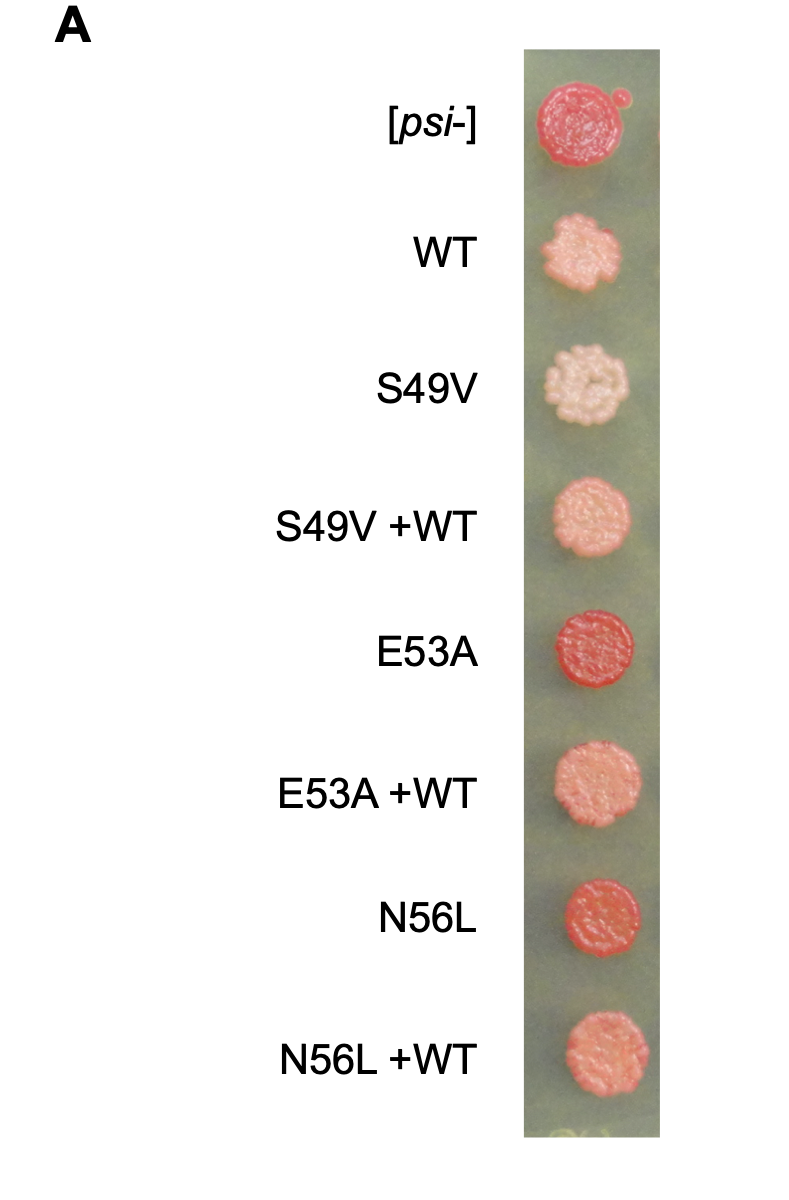

Supplement: S1 Fig — (A) sis1Δ [PSI+] strains expressing a single copy of wild-type or mutated SIS1, and strains expressing an additional copy of wild-type SIS1 (+WT) were spotted onto YPD media (n = 3). All spottings, minus the top (labeled [psi-]) are weak [PSI+] strains expressing the indicated constructs. (TIF) [file pone.0234207.s002.tif]

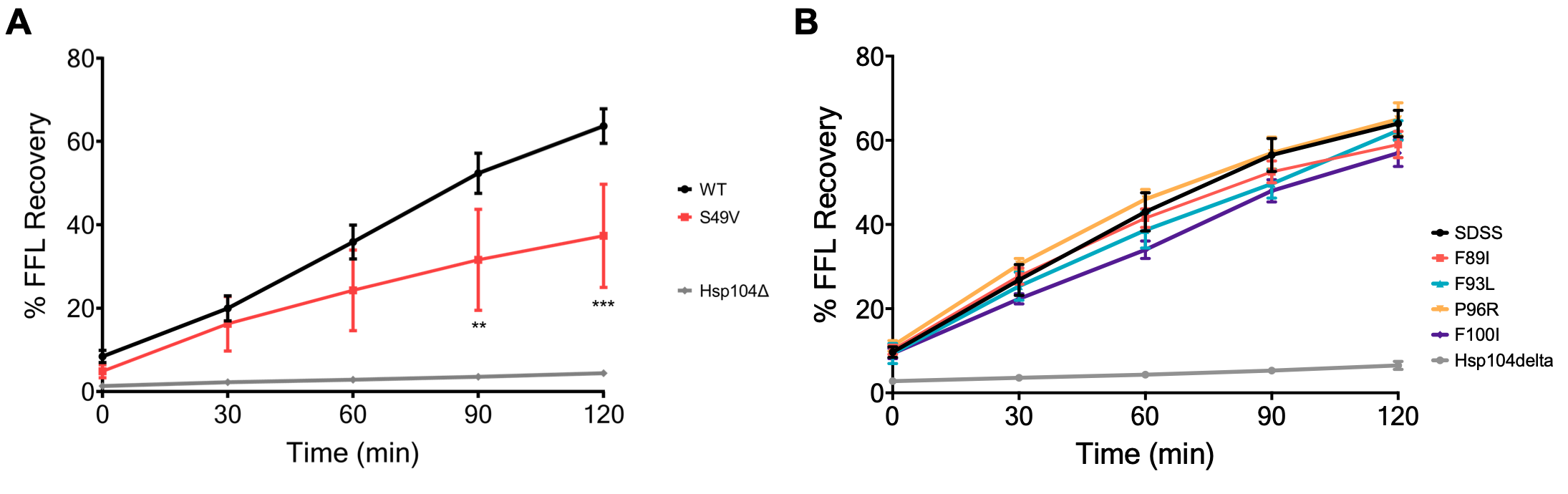

Supplement: S2 Fig — (A) the capability for refolding luciferase was measured in [rnq-] sis1Δ yeast strains harboring the indicated construct along with a plasmid expressing luciferase. (B) same as in A, but in Sc37 [PSI+] strains expressing wild-type or the mutated chimeric construct SDSS constructs instead of Sis1. Yeast were normalized, treated with cycloheximide and subjected to heat shock at 42°C for 22 minutes, followed by recovery at 30°C. Luminescence was measured at the indicated timepoints during recovery and normalized to luminescence of samples without heat shock treatment. The amount of luciferase refolding is plotted as percentage of recovery and represented as mean SEM (n = 6). (TIF) [file pone.0234207.s003.tif]

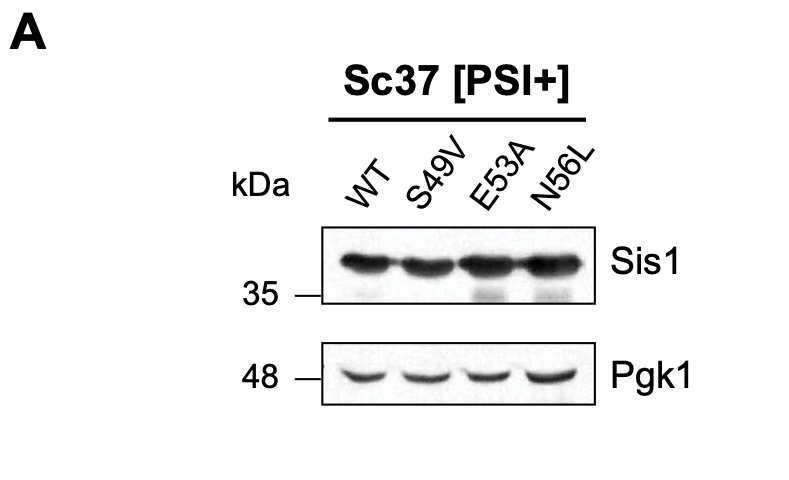

Supplement: S3 Fig — (A) Representative western blot showing expression of Sis1 in Sc37 [PSI+] sis1Δ yeast strains harboring the indicated construct (n = 3). Pgk1 is shown as a loading control. All samples were analyzed under the same experimental conditions. (TIF) [file pone.0234207.s004.tif]
